# Reactivation of Human Herpesvirus (HHV) 6 as Etiology of Acute Liver Injury in Drug Reaction With Eosinophilia and Systemic Symptoms (DRESS) Syndrome: A Case Report

**DOI:** 10.7759/cureus.29697

**Published:** 2022-09-28

**Authors:** Jonathan C Li

**Affiliations:** 1 Internal Medicine-Pediatrics Residency Program, University of Pittsburgh Medical Center, Pittsburgh, USA; 2 Internal Medicine-Pediatrics Residency Program, ChristianaCare, Newark, USA

**Keywords:** pediatrics, internal medicine, acute viral hepatitis, human herpesvirus-6 (hhv-6), drug reaction with eosinophilia and systemic symptoms (dress)

## Abstract

Drug reaction with eosinophilia and systemic symptoms (DRESS) syndrome is a rare drug-related disease. Key clinical components include fever, rash, eosinophilia, lymphadenopathy, hepatitis, and other end-organ damage. The pathophysiology of this disease is not fully understood. Viral reactivation has been implicated to be a component of the disease process. We report the case of a 21-year-old patient diagnosed with DRESS syndrome found to have the presence of human herpesvirus 6 (HHV 6) in both blood and biopsied liver tissue supporting viral hepatitis as the cause of liver injury in DRESS.

## Introduction

Drug reaction with eosinophilia and systemic symptoms (DRESS) syndrome is a rare disease with an estimated incidence of one in 1,000-10,000 drug exposures and up to a 10% mortality rate [1]. Although many drugs have been reported to cause DRESS, it is most commonly associated with the use of antiepileptic drugs, antibiotics, sulfa drugs, and allopurinol [1-3]. The etiology of this disease is not fully understood but is suspected to include: (i) accumulation of toxic drug metabolites triggering systemic inflammation, and (ii) drug-induced viral reactivation [1,2]. The most well-recognized viruses associated with DRESS are from the *Herpesviridae* family. These include Epstein-Barr virus (EBV), cytomegalovirus (CMV), human herpesvirus (HHV)-6, and HHV-7 [1,2,4]. Multiple criteria exist to aid in the diagnosis of DRESS [5]. Both Bocquet’s criteria and the Registry of Severe Cutaneous Adverse Reaction (RegiSCAR) appear to be equally effective in identifying DRESS [6-8]. An adaptation of RegiSCAR criteria by a Japanese consensus group further includes HHV-6 reactivation amongst its diagnostic criteria [9]. Here, we report the case of a 21-year-old patient diagnosed with DRESS syndrome who was found to have profound HHV-6 viremia and elevated liver enzymes with HHV-6 detected in biopsied liver tissue. 

## Case presentation

A previously healthy 21-year-old female was initiated on lamotrigine therapy for bipolar depression. Three weeks into treatment, she developed a sore throat and fever. Despite a negative rapid strep test and strep culture, her primary care physician treated her empirically with penicillin. One week later, she presented to the emergency department with persistent fever, new-onset rash, and vomiting with oral intake. She stated that her rash started on the left wrist and spread to her entire body over two days. She also complained of painful bilateral preauricular lymphadenopathy. Physical exam was significant for a pruritic, diffuse, red-purple maculopapular rash sparing the palms/soles/mucous membranes, tonsillar erythema with exudates, and right-cervical lymphadenopathy. Dermatology was consulted. They were concerned about early DRESS vs. viral exanthem. Repeat rapid strep testing was negative as was a mono-spot. Bloodwork was significant for an increased leukocyte count with a neutrophil predominance, normal eosinophil percent, and mildly increased aspartate transaminase (AST) and alanine transaminase (ALT) (Table 1). The patient was treated with IV fluids, acetaminophen, diphenhydramine, and ondansetron before being discharged home.

**Table 1:** Select laboratory values during the disease course AST: aspartate aminotransferase; ALT: alanine aminotransferase; ALP: alkaline phosphatase

	Reference	Day 1	Day 2	Day 4	Day 6	Day 10	Day 11	Day 12	Day 13	Day 14
Leukocyte count	2.5-11 /nL	15.3		21.6	29.7	22.8				14.5
Neutrophil %		86%		36%	39%	48%				36%
Eosinophil %		2%	1%	3%	12%	3%				
Lymphocyte %				31%	22%	32%				54%
Atypical lymphocyte %				10%	11%	0%				
AST	11-39 U/L	68		382	72	864	1,330	1,534	958	340
ALT	7-52 U/L	146		536	348	1,129	1,534	2,020	1,856	1,289
ALP	50-175 U/L			327	231	255				
Total bilirubin	0.2-1 mg/gL			3.7	1.2	4.9				
Lactate	0-2.2 mmol/L			3.5						
Ferritin	12-150 ng/mL						11,852			

The patient presented 24 hours after discharge for left-sided facial swelling involving the eyelid, cheek, and lips. Bloodwork was relatively unchanged from the prior test. Eosinophil count remained normal. She was started on IV methylprednisolone, admitted for 24-hour observation, and infectious disease was consulted. Differential diagnosis was most suspicious for viral exanthem vs drug rash. CMV IgM and EBV serologies (IgM, IgG, Epstein-Barr nuclear antigen (EBNA), early antigen (EA), polymerase chain reaction (PCR) quant) were obtained and were not supportive of acute infection. She was discharged home on a high-dose steroid taper. 

Two days later, she presented back to the emergency department with worsening facial rash with edema, vomiting, abdominal pain, diarrhea, fever, sore throat, dyspnea, and presyncope. She was noted to have low systolic blood pressures of 90-100 mmHg and a waxing and waning mental status. The dermatologic exam was significant for a non-blanching violaceous rash diffusely involving the face, trunk, and extremities, with a greater confluence in the flexural creases, and lesions involving the palms and the soles. Complete blood count (CBC) was significant for an increased leukocytosis that was notably different with lymphocyte predominance, presence of atypical lymphocytes, and a normal eosinophil count. Blood chemistries revealed elevated lactate and liver function tests (LFTs). Broad-spectrum antibiotics (IV) vancomycin, imipenem, and doxycycline were initiated. Steroids were continued. Throat, urine, and blood cultures were obtained and ultimately negative for infection. A respiratory pathogen panel was negative. Reassessment by dermatology at this time favored viral exanthem over cutaneous drug reaction based on rash morphology, a biopsy was deferred by patient request. She remained in the ICU for 48 hours, during this time her rash improved dramatically as did her systemic symptoms. Repeat CBC revealed worsened leukocytosis with prominent eosinophilia and persistent presence of atypical lymphocytes. LFTs had improved. She was discharged home on a steroid taper.

**Figure 1:**
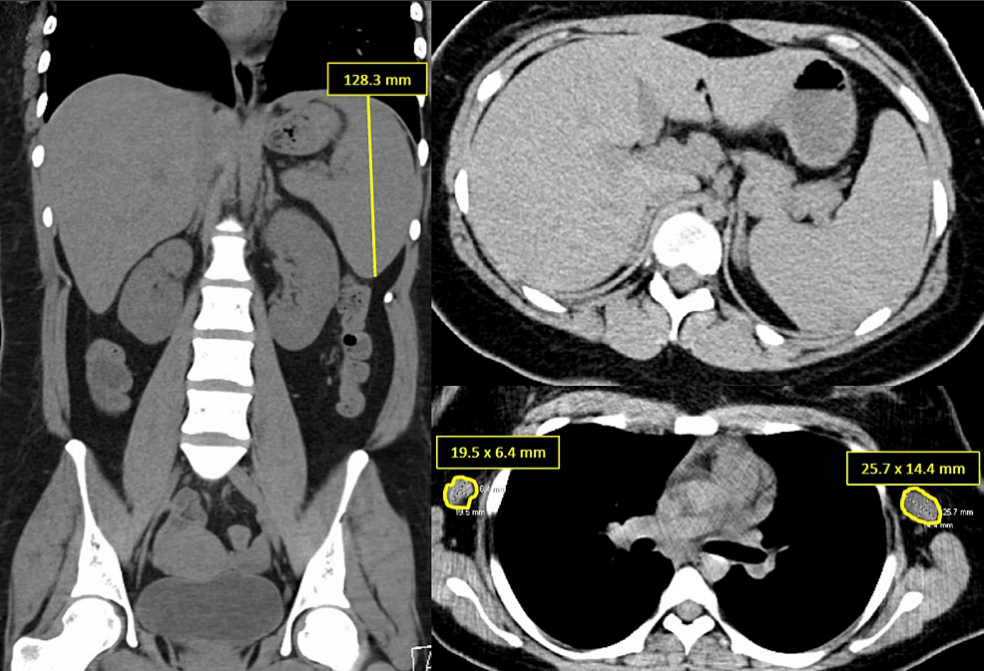
CT of the chest, abdomen, and pelvis revealing splenomegaly and axillary lymphadenopathy

Four days later, the patient presented again to the ED after experiencing multiple fevers overnight to 103.6F with episodes of nausea and vomiting. As per the patient and family, the rash had not worsened since discharge from the ICU, and the skin was described as diffusely erythrodermic by the admitting physician. CBC at this time revealed a persistent leukocytosis with neutrophil predominance, no atypical lymphocytes, and resolution of eosinophilia. LFTs were even more elevated than prior hospitalization (>20 times the upper limit normal with an AST:ALT ratio of 0.76). Right upper quadrant ultrasound revealed borderline hepatomegaly, splenomegaly, and coarsened liver echotexture suggestive of underlying liver disease. HIV, viral hepatitides, herpes simplex virus (HSV) 1/2, HHV-6, repeat CMV, repeat EBV, West Nile virus, and acetaminophen studies were obtained. IV acyclovir was started empirically. Blood cultures were obtained again and empiric antibiotics were initiated. CT abdomen and pelvis revealed hepatosplenomegaly without focal lesions and bilateral axillary lymphadenopathy overall concerning for a systemic disease such as hematologic malignancy (Figure 1).

**Figure 2:**
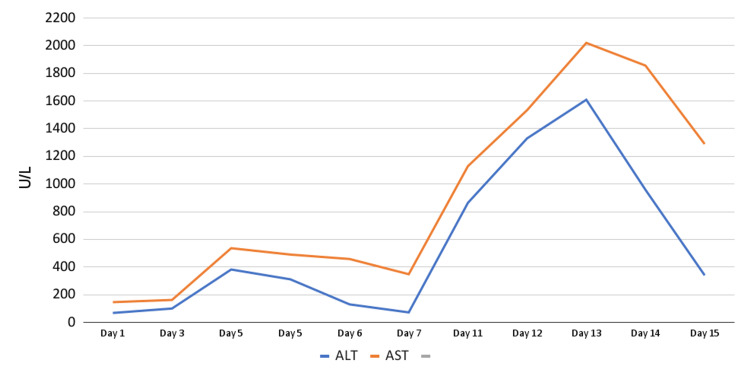
Elevated transaminase trend AST: aspartate transaminase; ALT: alanine aminotransferase

**Figure 3:**
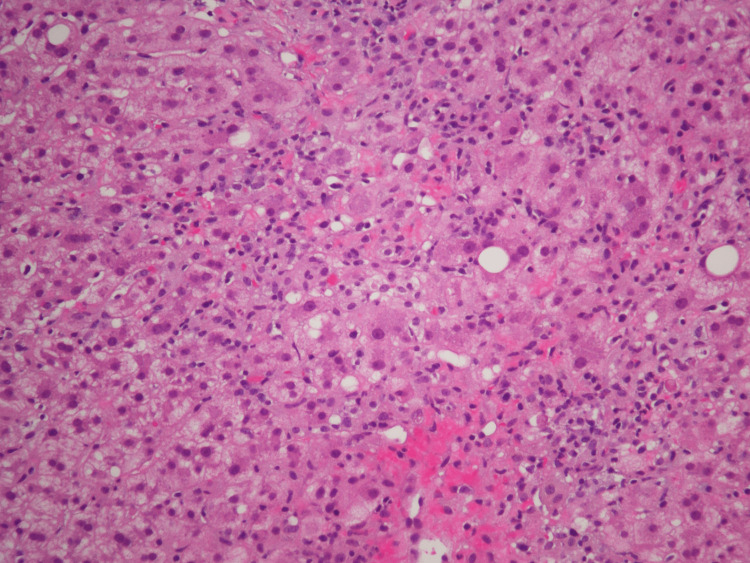
Liver biopsy (H&E Stain) H&E: hematoxylin and eosin

On day two, AST and ALT continued to rise. Gastroenterology was consulted and recommended workup for Wilson’s disease and autoimmune hepatitis, Doppler ultrasound to rule out portal vein thrombosis, and liver biopsy due to concern for lymphoma. Hematology/oncology was also consulted and agreed with obtaining a biopsy. Ferritin was obtained due to concern for hemophagocytic lymphohistiocytosis (HLH) and was found to be elevated but not enough to support a diagnosis of HLH. The hematology/oncology consultants did not feel the clinical picture was severe enough for HLH either. On day three, AST and ALT peaked and subsequently began to trend down (Figure 2). A core liver biopsy was obtained on hospital day four (Figure 3).

The primary pathology report showed severe acute hepatitis with lobular inflammation and focal confluent necrosis. Iron stain was not significant, ruling out hemochromatosis. Periodic acid-Schiff (PAS) stain did not suggest a glycogen storage disease. Trichrome stain did not show fibrosis. A second report was obtained from a major academic medical center in which they reported lymphocyte predominant lobular inflammation with some plasma cells, rare eosinophils, and scattered macrophages overall consistent with severe acute hepatitis. They performed a CMV stain, which was negative, and given the clinical information provided, they agreed that medication-induced injury should be the top differential. They also commented that in the literature, HHV-6-induced liver injury in immunosuppressed patients such as stem cell or liver transplants, tend to have a pattern of injury that appears to be periportal rather than centrilobular.

On day five, cell counts and LFTs continued to improve. The patient was discharged home on a prednisone taper. Serum quantitative PCR for HHV-6 resulted with a DNA copy load of >2,000,000 copies/mL (ref: <500 copies/mL). Tissue HHV-6 PCR also resulted in an abnormal result but the quantity was not reported by the lab. Since discharge, the patient was followed by dermatology and had continued improvement in rash and liver function tests.

## Discussion

DRESS syndrome is a rare and potentially life-threatening condition. Symptoms typically start two to six weeks after initiation of medication and usually include fevers, facial edema, and rash [10]. Patients have a high likelihood of developing end-organ damage, most commonly manifested as liver failure, though severe pulmonary and renal damage have been reported, and mortality can reach 10-20% [11]. A drug-induced hypersensitivity reaction, most often from anticonvulsant medications like lamotrigine, plays an integral role in the pathogenesis of DRESS syndrome. Viral reactivation from the *Herpesviridae* family is also known to contribute to pathogenesis and likely influences the overall severity of the illness. Although not entirely understood, it is thought that viremia has direct cytotoxic effects or causes an immune response that results in tissue damage [4]. Diagnosis is clinical and there are multiple criteria currently published to assist with this (Table 2). Management of DRESS syndrome includes discontinuation of the offending drug and the addition of steroids in severe disease. Specific antiviral therapy is rarely used, in part due to the toxicity of the agents. Recovery involves a relapsing and remitting course, which can often take weeks to months. 

**Table 2:** Comparative table of diagnostic criteria for DRESS syndrome *Three out of four asterisked (*) criteria are required for making the diagnosis. RegiSCAR: Registry of Severe Cutaneous Adverse Reaction

Bocquet’s criteria [6]	RegiSCAR criteria [7]	Japanese consensus group criteria for atypical Drug-Induced Hypersensitivity Syndrome (DiHS) [9]
1) Skin eruption	1) Hospitalization	1) Maculopapular rash developing >3 weeks after starting with the suspected drug
2) Blood eosinophilia (>1.5×10^3^/µL) or the presence of atypical lymphocytes	2) Reaction suspected to be drug-related	2) Prolonged clinical symptoms 2 weeks after discontinuation of the suspected drug
3) Internal organ involvement, including lymphadenopathies (>2 cm in diameter), hepatitis (liver transaminases values > twice the upper normal limit), interstitial nephritis, and interstitial pneumonia or carditis.	3) Acute rash	3) Fever >38°C
	4) Fever >38°C*	4) Liver abnormalities (alanine aminotransferase>100U/L)
	5) Enlarged lymph nodes at a minimum of 2 sites*	5) Leukocyte abnormalities: Leukocytosis (>11 X 10^9^/L), Atypical lymphocytosis (>5%) or Eosinophilia (>1.5 x 10^9^ /L)
	6) Involvement of at least 1 internal organ*	6) Lymphadenopathy
	7) Blood count abnormalities*: Lymphocytes above or below normal limits, Eosinophils above the laboratory limits, Platelets below the laboratory limits	7) Human Herpes 6 reactivation
		The diagnosis is confirmed by the presence of five of the seven criteria (typical DiHS).

As reviewed by Pantry and Medveczky, HHV-6 represents two distinct viruses HHV-6A and HHV-6B. Both are lymphotropic viruses that selectively infect T-lymphocytes, with a preference toward CD4+ T-cells. Almost all people are seropositive for HHV-6. HHV-6B is the predominant cause of pediatric infection by age two causing roseola infantum in the United States. Studies have shown that lifelong latency from HHV-6 occurs within monocyte/macrophage cell lines by integrating into chromosomal telomeres. This can affect germline cells and approximately 1% of the global population has inherited chromosomally integrated HHV-6. These individuals exhibit a persistent high viral load [12]. 

Apart from DRESS, one of the most well-studied clinical scenarios for HHV-6 reactivation is among liver transplant patients. As reviewed by Phan et al., HHV-6 reactivation among this population leads to fever, hepatitis, and encephalitis. The most commonly identified virus is HHV-6B. Histologically, they report that portal lymphocytic infiltration and periportal necrosis are suggestive of HHV-6. Localization of HHV-6 within the liver has been reported to be sinusoidal mononuclear cells and the nuclei of intrahepatic bile duct epithelial cells. Risk factors for HHV-6 reactivation include corticosteroids and T-cell defects. Currently, there are no FDA-approved medications for treating HHV-6 infection. In vitro studies and clinical reports suggest gancyclovir, cidofovir, and foscarnet are effective antiviral treatments [13].

The relationship between HHV-6 reaction and DRESS syndrome is not fully understood but likely includes a complex interplay between drug metabolism, immune activation, and genetic predisposition as discussed earlier. Though various members of the *Herpesviridae* family have been associated with DRESS, the most common virus is HHV-6. Ishida et al. evaluated Stevens-Johnson syndrome (SJS), toxic epidermal necrolysis (TEN), and drug-induced hypersensitivity syndrome (DIHS)/DRESS and found that compared to EBV and CMV, HHV-6 is specific for DIHS/DRESS [14]. Currently, the management of DRESS is focused on the withdrawal of the culprit drug and supportive care. Systemic corticosteroids are recommended for severe cases (internal organ dysfunction). Clinical reports suggest steroids may be unnecessary for mild cases [15]. However, since systemic steroids are a risk factor for HHV-6 reactivation among liver transplant patients, their use in managing DRESS becomes less clear. Although necessary to suppress systemic inflammation, they may provoke worsening viremia. If viral reactivation is confirmed, there may be a role for antiviral therapy to quell viral replication, but the evidence for the use of antivirals in managing DRESS has yet to be determined. 

In DRESS, the most commonly affected organ apart from the skin is the liver [3,16,17] Though liver histology is well-reported for DRESS, this is the first report, to our knowledge, to suggest HHV-6 reactivation within the liver parenchyma itself [1,3,17-20]. However, unlike the histologic patterns reported for HHV-6 reactivation among liver transplant patients, we observed centrilobular necrosis and normal portal tracts, bile ducts, and vessels. Fujita et al. similarly reported bridging perivenular necrosis and infiltration of the lymphocytes and eosinophils [18]. A series of liver biopsies (n=7) performed by Ichai et al. in the setting of liver injury during DRESS found that all cases had intralobular necrosis, most commonly centrilobular necrosis (n=3), and lymphocytic infiltrate. Eosinophilic infiltrate was seen in five cases and biliary involvement in four [20]. We suspect the mode of reactivation in DRESS is different than that in liver transplant and, therefore, reveals a distinctly different histologic pattern. This may be an important distinction for clinical pathologists.

## Conclusions

Recognition of herpes virus reactivation in DRESS is growing as is their suspected role in the disease's pathogenesis. Of these viruses, HHV-6 is commonly implicated. Liver injury is a salient clinical feature of DRESS. We reported a case of lamotrigine-induced DRESS with severe hepatitis and significant HHV-6 viremia where a liver biopsy was performed. PCR of hepatic parenchyma was also positive for HHV-6, suggestive of viral reactivation as the cause of hepatitis in DRESS. To our knowledge, this is the first case of DRESS to report concomitant detection of HHV-6 in liver parenchyma with viremia.
